# A Mismatch between High-Risk Behaviors and Screening of Infectious Diseases among People Who Inject Drugs in Dar es Salaam, Tanzania

**DOI:** 10.1371/journal.pone.0148598

**Published:** 2016-02-05

**Authors:** Linda Beatrice Mlunde, Bruno Fokas Sunguya, Jessie Kazeni Mbwambo, Omary Said Ubuguyu, Akira Shibanuma, Junko Yasuoka, Masamine Jimba

**Affiliations:** 1 Department of Community and Global Health, Graduate School of Medicine, The University of Tokyo, 7-3-1, Hongo, Bunkyo-ku, Tokyo, 113–0033, Japan; 2 Department of Community Health, School of Public Health and Social Sciences, Muhimbili University of Health and Allied Sciences, P.O. Box 65015, Dar es Salaam, Tanzania; 3 Department of Psychiatry and Mental Health, Muhimbili National Hospital, P.O. Box 65000, Dar es Salaam, Tanzania; University of Athens, Medical School, GREECE

## Abstract

**Background:**

People who inject drugs are at risk of various infectious diseases. Despite such a risk, evidence is limited which studied the utilization of screening services for common infectious diseases among people who inject drugs in Tanzania. We aimed to examine their high-risk behaviors; utilization of screening services for HIV infection, hepatitis B/C, any other sexually transmitted infection, and tuberculosis; and their associated factors in Dar es Salaam, Tanzania.

**Methods:**

We conducted a baseline cross-sectional study as part of a prospective cohort study of people who inject drugs. We included 578 participants comprising of new enrollees of the integrated methadone-assisted treatment program and those who were selected from the communities but not enrolled in the program. We interviewed new enrollees preceding their enrollment and receipt of services from the program. We measured participants’ high-risk behaviors and their utilization of screening services. We analyzed the data descriptively and used multiple logistic regressions to identify the factors associated with ever being screened for infectious diseases.

**Results:**

Of 578 participants, 14.2% shared injection needles. Of 547 sexually active participants, 37.5% had multiple sexual partners and only 17.4% used a condom. Of all participants, however, only 36.0% had ever been screened for HIV infection, 18.5% for tuberculosis, 11.8% for any other sexually transmitted infection, and 11.6% for hepatitis B/C. They were more likely to have ever been screened for HIV infection if they had education levels above primary education (adjusted odds ratio [AOR]: 2.54, 95% CI: 1.54–4.20), had a history of transactional sex (OR: 2.63, 95% CI: 1.01–6.84), and were new enrollees of the program (AOR: 7.41, 95% CI: 4.41–12.86).

**Conclusions:**

People who inject drugs practice high-risk behaviors but their utilization of screening services for infectious diseases is poor in Dar es Salaam, Tanzania. It is crucial to increase the coverage of screening services for them and strengthen the counseling of safer sexual practices.

## Introduction

People who inject drugs are at risk of various infectious diseases [1–4]. They engage in high-risk injecting and sexual behaviors that predispose them to HIV infection, hepatitis, and other sexually transmitted infections (STIs) [5–8]. Once they are infected with one infectious disease, they may be at an increased risk of another or progress the pathogenesis of another. For example, HIV-positive people who inject drugs are at a high risk of tuberculosis [9, 10]. While STIs increase the risk of HIV infection [11], HIV infection accelerates the progress of viral hepatitis [12, 13].

The World Health Organization recommends routine voluntary HIV testing and counseling, screening of STIs, hepatitis B/C, and tuberculosis to all key populations including people who inject drugs [14]. Screening interventions can mitigate the transmissions of these infectious diseases. If individuals know their infection statuses, they may engage in safer sexual [15] and injecting behaviors [16]. Many people who inject drugs, however, do not know their infection statuses [17]. Also, they often receive screening services late and fail to receive care.

Injecting drug use is a growing problem in Tanzania–a hot spot for major drug trafficking routes in Africa [18]. People who inject drugs also engage in high-risk injecting and sexual behaviors in Tanzania [19] and they are at risk of infectious diseases. It is important that this key population gets access to the available services for screening infectious diseases in the country [19, 20]. Early diagnosis of STIs is one of the objectives of the Tanzanian HIV infection policy [21]. Most men and women, however, do not have STIs symptoms despite being infected, or show mild symptoms making them not seek care [21]. Also, knowledge of STIs is low in the country [22]. The policy recommends screening of STIs to all patients when they seek care for other medical problems. The commonly screened STIs include syphilis, gonorrhea, chlamydia, and trichomoniasis [23]. Anecdotal evidence shows that only youth can receive free screening services for STIs in the STI clinics, however, adults have to pay for them.

Evidence is limited on whether people who inject drugs use screening services for infectious diseases appropriately. Tanzania has about 30,000 people who inject drugs and Dar es Salaam is estimated to hold 30–50.0% of them [24]. HIV prevalence among people who inject drugs stands at 35.0% in the country [24], a seven-times fold to that of the general population. Despite such burden, only 47.6% and 2.0% of them were screened for HIV infection and Hepatitis C respectively in Temeke, one of the three municipalities of the Dar es Salaam region [25]. Evidence is not available about the utilization of screening services for other STIs and tuberculosis despite their high burdens in the country [26–28]. In the current study, we examined the magnitude of high-risk behaviors, the utilization of screening services for HIV infection, hepatitis B/C, any other STI, and tuberculosis, and their associated factors among people who inject drugs in all three municipalities of Dar es Salaam, Tanzania.

## Methods

### Study design and participants

We conducted this cross-sectional study as a baseline phase of a prospective cohort study of people who inject drugs in Dar es Salaam, Tanzania. The three municipalities of the region include Kinondoni, Ilala, and Temeke. The aim of the cohort study was to the examine the roles of an integrated methadone-assisted treatment program in reducing high-risk injecting and sexual behaviors, criminal activities, and improving health care seeking behavior among people who inject drugs. We included people who inject drugs who were new enrollees of the integrated methadone-assisted treatment program. We also included community-recruited people who inject drugs who were not enrolled in the program. The inclusion criteria were: using illicit drugs by injection method at least once in the past 30 days, being 18 years or older, and being a resident of Dar es Salaam. Having a severe mental illness was the exclusion criterion. We assessed mental health status based on presence or absence of gross disorganization in behavior and speech and being cognizant of time, place, and person. None had a severe mental illness, among people who inject drugs we approached.

We recruited participants using convenience sampling. First, we recruited new enrollees of the integrated methadone-assisted treatment program from the hospitals that provided the program’s services at the time of data collection. Only two hospitals were providing these services: Muhimbili National Hospital in Ilala municipality and Mwananyamala Hospital in Kinondoni municipality. Research assistants approached new enrollees and invited them to participate in the study. We followed this process until the sample size was attained.

Second, we used a snowball sampling to recruit people who inject drugs from the communities of all three municipalities in Dar es Salaam. We recruited initial participants assisted by former people who inject drugs and were receiving treatment at the integrated methadone-assisted treatment program’s clinics. They introduced us to people who inject drugs in the locations where they usually meet to use drugs. After completing the interviews, we requested the interviewed people who inject drugs to give us directions or one of them to take us to other places where we could recruit other people who inject drugs. We repeated this procedure until the expected sample size was attained

### Data collection

We used a structured pre-tested questionnaire in Swahili to conduct face-to-face interviews for data collection. We conducted this baseline survey from January to April 2014. We interviewed new enrollees of the integrated methadone-assisted treatment program in the consultation rooms of the program’s clinics. The program’s enrollees receive various services at these clinics including methadone [19], medical care, voluntary screening of various infectious diseases [29, 30], and psychosocial counseling. We interviewed them about their behaviors preceding enrollment and receipt of services from the integrated methadone-assisted treatment program.

We conducted interviews with community-recruited people who inject drugs at places where people who use drugs usually meet to inject drugs. We selected a location that was far from the rest of other people who inject drugs to conduct the interview. We approached a total of 600 people who inject drugs (280 new enrollees and 320 community-recruited ones); only two of them refused to participate.

### Measurements

The outcome variables were ever been screened for HIV infection, hepatitis B/C, any other STI, and tuberculosis. We assessed participants’ screening statuses for such infectious diseases using modified questions from the instruments used to assess the health status of the enrollees of the program. We asked them whether they had ever been screened for HIV infection, hepatitis B/C, any other STI, and tuberculosis.

Independent variables included injecting and sexual behaviors and polysubstance use. We assessed participants’ injecting behaviors using modified questions from the program’s health status assessment instruments. We asked them whether they had practiced high-risk injecting behaviors such as sharing injecting needles [19] and engaging in flashblood—a practice of a sharing syringe filled with blood drawn from an individual who had just injected drugs [31]. We assessed their high-risk sexual behaviors such as unprotected sex and multiple sexual partnerships using questions extracted from the Tanzania Demographic and Health Survey questionnaire [32]. We measured polysubstance use by asking them whether they had used more than one type of substance in the past 30 days. Examples of such substances included heroin, cannabis, stimulants, and sedatives. We adapted the questions from the program’s health status assessment instruments.

We also measured participants’ history of imprisonment and sociodemographic characteristics as independent variables. We asked them if they had a lifetime history of being imprisoned. The sociodemographic characteristics included questions on age, sex, marital status, education level, and employment status. We adapted such questions from the Addiction Severity Index (ASI) fifth edition [33]. The ASI is also used to screen program’s enrollees prior to and during treatment at the integrated methadone-assisted treatment program’s clinics [19]. We used similar instruments for new enrollees and community-recruited people who inject drugs.

### Statistical analysis

We analyzed baseline data of 578 participants together (273 new enrollees and 305 community-recruited people who inject drugs). Out of 598 responses, we excluded 20 (7 for new enrollees and 13 for community-recruited ones) due to missing data. Participants of the excluded and included questionnaires had similar age, education level, employment status, and marital status.

We compared the characteristics of participants who had and those who had not ever been screened for HIV infection, hepatitis B/C, any other STI, and tuberculosis using t-tests and chi-squared tests. Then, we conducted bivariate logistic regression analyses followed by multiple logistic regression analyses to identify the factors associated with ever being screened for each of the above-mentioned infections. We assessed the interaction terms of all the independent variables with the integrated methadone-assisted treatment program enrollment status variable. We categorized it into new enrollees and community-recruited people who inject drugs. Only the interaction term of condom use at the last sexual encounter and enrollment status was statistically significant for the model of hepatitis B/C screening. The interaction term of age and enrollment status was the only one statistically significant for the model of any other STI screening. None of the interaction terms were statistically significant for the models of HIV infection and tuberculosis screening. The goodness-of-fit tests were satisfactory for all four models: HIV infection—p = 0.519, hepatitis B/C—p = 0.867, any other STI—p = 0.882, and tuberculosis—p = 0.282. We adjusted for all the independent variables in the regression models to avoid the risk of over-fitting [34]. We set the statistical significance level at p<0.05, and used STATA 12 software for all analyses.

### Ethical considerations

Before the interview, we explained to the participants the contents of the study, its procedures, and consent form. We also explained to them that their participation would not affect their treatment at the integrated methadone-assisted treatment program’s clinics (for new program enrollees) or have legal implications. Then those who gave verbal consent to participate were recruited into the study and signed the written consent forms. Participation was voluntary. Privacy and confidentiality were ensured. Participants were reimbursed 1000 Tanzanian shillings (about 0.63 US dollars) for their participation. The study procedures were reviewed and approved by the Research Ethics Committee of The University of Tokyo and the Senate Research and Publications Committee of Muhimbili University of Health and Allied Sciences.

## Results

### General characteristics of participants

Table 1 shows the sociodemographic characteristics of all participants. In total, 578 people who inject drugs participated in this study. Their mean age was 34.6 years (standard deviation [SD] 5.7). About 95% of them were male. Among those who had ever been screened (n = 260), 33.1% had education levels higher than primary education compared with 7.9% of those who had not ever been screened (n = 318) (p<0.001). More participants who had ever been screened were married compared with those who had not ever been screened (18.5% vs. 6.9%, p<0.001). Also, more participants who had ever been screened had employment compared with those who had not ever been screened (79.2% vs. 63.5%, p<0.001).

**Table 1:** Sociodemographic characteristics and history of imprisonment of participants.

Variable	Total	Screened for infections ^a^	Not screened for infections ^a^	p value
	(n = 578)	(n = 260)	(n = 318)	
	n (%)	n (%)	n (%)	
Age mean (SD^b^)	34.6 (5.7)	34.2 (6.6)	34.9 (4.9)	0.153
Sex				
Male	548 (94.8)	244 (93.9)	304 (95.6)	0.345
Female	30 (5.2)	16 (6.1)	14 (4.4)	
Educational level				
Primary or lower	467 (80.8)	174 (66.9)	293 (92.1)	<0.001
Higher than primary	111 (19.2)	86 (33.1)	25 (7.9)	
Marital status				
Married	70 (12.1)	48 (18.5)	22 (6.9)	<0.001
Unmarried	508 (87.9)	212 (81.5)	296 (93.1)	
Employment				
Yes	408 (70.6)	206 (79.2)	202 (63.5)	<0.001
No	170 (29.4)	54 (20.8)	116 (36.5)	
Income per day in US $, mean (SD^b^)	11.2 (14.9)	9.5 (18.1)	12.5 (11.5)	0.017
History of imprisonment				
Yes	152 (26.3)	66 (25.4)	86 (27.0)	0.652
No	426 (73.7)	194 (74.6)	232 (73.0)	
MAT program enrollment status				
New MAT^c^ program enrollee	273 (47.2)	194 (74.6)	79 (24.8)	<0.001
Community-recruited PWID^d^	305 (52.8)	66 (25.4)	239 (75.2)	

^a^ Infections: HIV, hepatitis B/C, any other STI, tuberculosis.

^b^SD: standard deviation.

^c^MAT: integrated methadone-assisted treatment.

^d^PWID: people who inject drugs

### Utilization of screening services for infectious diseases by participants

Of all participants (n = 578), 36.0% had ever been screened for HIV infection, 18.5% for tuberculosis, 11.8% for any other STI, and 11.6% for hepatitis B/C. Among all the participants, 45.0% had ever been screened for any of the above-mentioned infections (results not shown).

### Injecting and sexual behaviors of participants

Table 2 shows injecting and sexual behaviors of all participants. Of 578, 14.2% shared a needle at the last injection. Among those who had ever been screened, 19.6% reported it compared with 9.8% of those who had not ever been screened (p = 0.001). In addition, of all participants, 15.6% had ever practiced flashblood. Among those who had ever been screened, 9.2% reported it compared with 20.8% of those who had not ever been screened (p<0.001). Moreover, of all participants, 6.8% had ever had transactional sex. Among those who had ever been screened, 10.4% reported it compared with 3.8% of those who had not ever been screened (p = 0.002).

**Table 2:** Injecting and sexual behaviors of participants.

Variable	Total	Screened for infections^a^	Not screened for infections^a^	
	(n = 578)	(n = 260)	(n = 318)	
	n (%)	n (%)	n (%)	p-value
Sharing needle at the last injection				
Yes	82 (14.2)	51 (19.6)	31 (9.8)	0.001
No	496 (85.8)	209 (80.4)	287 (90.2)	
Ever practiced flashblood				
Yes	90 (15.6)	24 (9.2)	66 (20.8)	<0.001
No	488 (84.4)	236 (90.8)	252 (79.2)	
Ever had transactional sex				
Yes	39 (6.8)	27 (10.4)	12 (3.8)	0.002
No	539 (93.2)	233 (89.6)	306 (96.2)	
Anal sex in the past 6 months ^b^				
Yes	58 (10.6)	23 (9.6)	35 (11.4)	0.493
No	489 (89.4)	217 (90.4)	272 (88.6)	
Multiple sexual partners ^b^				
Yes	205 (37.5)	74 (30.8)	131 (42.7)	0.005
No	342 (62.5)	166 (69.2)	176 (57.3)	
Condom use at last sex ^b^				
Yes	95 (17.4)	68 (28.3)	27 (8.8)	<0.001
No	452 (82.6)	172 (71.7)	280 (91.2)	
Polysubstance use				
Yes	323 (55.9)	137 (52.7)	186 (58.5)	0.163
No	255 (44.1)	123 (47.3)	132 (41.5)	

^a^ Infections: HIV, hepatitis B/C, any other STI, tuberculosis.

^b^ Among sexually active participants in the past six months, n = 547.

### Factors associated with ever being screened for HIV infection among participants

Table 3 shows the results of regression analyses. People who inject drugs were more likely to have ever been screened for HIV infection when they had education levels higher than primary education (adjusted odds ratio [AOR]: 2.54, 95% Confidence Interval [CI]: 1.54–4.20), had a history of transactional sex (AOR: 2.63, 95% CI: 1.01–6.84). They were also more likely to have ever been screened for HIV infection when they were new enrollees of the integrated methadone-assisted treatment program (AOR: 7.41, 95% CI: 4.26–12.86).

**Table 3:** Factors associated with ever being screened for HIV among participants.

Variable	OR^a^	95% CI	p-value	AOR^b^	95% CI	p-value
Age	0.99	0.96–1.02	0.591	1.01	0.98–1.05	0.505
Sex						
Male	1.00			1.00		
Female	1.60	0.76–3.34	0.214	1.05	0.37–2.96	0.927
Educational level						
Primary or lower	1.00			1.00		
Higher than primary	4.72	3.04–7.34	<0.001	2.54	1.54–4.20	<0.001
Employment						
No	1.00			1.00		
Yes	2.09	1.40–3.12	<0.001	1.05	0.61–1.80	0.872
Income per day	0.93	0.85–1.02	0.140	1.03	0.93–1.13	0.586
Shared needle at the last injection						
No	1.00			1.00		
Yes	2.21	1.38–3.55	0.001	1.73	0.99–3.03	0.055
Ever practiced flashblood						
No	1.00			1.00		
Yes	0.36	0.21–0.63	<0.001	0.79	0.38–1.64	0.529
Ever had transactional sex						
No	1.00			1.00		
Yes	1.97	1.02–3.77	0.042	2.63	1.01–6.84	0.047
Anal sex in the past 6 months						
No	1.00			1.00		
Yes	0.70	0.38–1.26	0.228	0.80	0.36–1.76	0.581
Multiple partners						
No	1.00			1.00		
Yes	0.61	0.42–0.88	0.008	0.86	0.53–1.39	0.528
Condom use at last sexual encounter						
No	1.00			1.00		
Yes	2.92	1.90–4.49	<0.001	1.16	0.70–1.92	0.553
Polysubstance use in the past 30 days						
No	1.00			1.00		
Yes	0.71	0.51–1.00	0.050	0.84	0.54–1.29	0.428
History of imprisonment						
No	1.00			1.00		
Yes	0.90	0.61–1.33	0.595	1.55	0.93–2.56	0.090
MAT^c^ program enrollment status						
Community-recruited PWID^d^	1.00			1.00		
New enrollees	8.92	5.98–13.33	<0.001	7.41	4.26–12.86	<0.001

^a^OR: odds ratio.

^b^AOR: adjusted odds ratio.

^c^MAT: integrated methadone-assisted treatment.

^d^PWID: people who inject drugs.

All variables were included in the final regression model.

### Factors associated with ever being screened for hepatitis B/C among participants

Table 4 shows the results of regression analyses. People who inject drugs were more likely to have ever been screened for hepatitis B/C when they had a history of transactional sex (AOR: 5.30, 95% CI: 1.86–15.05), had a history of imprisonment (AOR: 4.15, 95% CI: 2.19–7.86), or used a condom at the last sexual encounter (AOR: 6.09, 95% CI: 1.45–25.58). They were also more likely to have ever been screened for hepatitis B/C when they were new enrollees of the methadone-assisted treatment program (AOR: 6.87, 95% CI: 2.69–17.53). The interaction of being both new enrollees and using condoms at the last sexual encounter was positively associated with ever being screened for hepatitis B/C (AOR: 7.23, 95% CI: 2.71–19.24). However, participants were less likely to have ever been screened for hepatitis B/C when they were employed (AOR: 0.34, 95% CI: 0.17–0.69).

**Table 4:** Factors associated with ever being screened for hepatitis B/C among participants.

Variable	OR^a^	95% CI	p-value	AOR^b^	95% CI	p-value
Age	0.97	0.92–1.01	0.116	0.97	0.93–1.02	0.265
Sex						
Male	1.00			1.00		
Female	2.00	0.78–5.08	0.147	0.46	0.12–1.68	0.239
Educational level						
Primary or lower	1.00			1.00		
Higher than primary	2.51	1.44–4.37	0.001	1.50	0.79–2.84	0.211
Marital status						
Unmarried	1.00			1.00		
Married	0.98	0.45–2.15	0.964	0.76	0.32–1.81	0.529
Employment						
No	1.00			1.00		
Yes	0.62	0.36–1.05	0.075	0.34	0.17–0.69	0.003
Income per day	1.00	0.90–1.11	0.982	0.96	0.83–1.10	0.559
Shared needle at the last injection						
No	1.00			1.00		
Yes	1.37	0.70–2.70	0.355	0.78	0.36–1.70	0.538
Ever practiced flashblood						
No	1.00			1.00		
Yes	0.82	0.39–1.73	0.608	1.30	0.49–3.48	0.600
Ever had transactional sex						
No	1.00			1.00		
Yes	3.91	1.88–8.16	<0.001	5.30	1.86–15.05	0.002
Anal sex in the past 6 months						
No	1.00			1.00		
Yes	1.65	0.79–3.45	0.179	1.16	0.43–3.11	0.770
Multiple partners						
No	1.00			1.00		
Yes	1.02	0.60–1.73	0.949	0.93	0.47–1.83	0.832
Polysubstance use in the past 30 days						
No	1.00			1.00		
Yes	0.56	0.34–0.94	0.029	0.68	0.38–1.23	0.205
History of imprisonment						
No	1.00			1.00		
Yes	2.78	1.65–4.68	<0.001	4.15	2.19–7.86	<0.001
Condom use*MAT^c^ program enrollment status						
Condom use	2.26	1.28–3.97	0.005	6.09	1.45–25.58	0.014
New enrollees	2.36	1.38–4.02	0.002	6.87	2.69–17.53	<0.001
Condom use*new enrollees	1.85	1.003–3.42	0.049	7.23	2.71–19.24	<0.001

^a^OR: odds ratio.

^b^AOR: adjusted odds ratio.

^c^MAT: integrated methadone-assisted treatment.

All variables were included in the final regression model.

### Factors associated with ever being screened for sexually transmitted infections among participants

Table 5 shows the results of regression analyses. People who inject drugs were more likely to have ever been screened for any other STI when they had a history of transactional sex (AOR: 3.54, 95% CI: 1.26–9.96) or used condom at the last sexual encounter (AOR: 1.92, 95% CI: 1.05–3.52. Those with older age were less likely to have ever been screened for any other STI (AOR: 0.87, 95% CI: 0.79–0.96). The interaction of being both older and a new enrollee of the integrated methadone-assisted treatment program, however, was positively associated with ever being screened for any other STI (AOR: 1.14, 96% CI: 1.02–1.27).

**Table 5:** Factors associated with ever being screened for sexually transmitted infections among participants.

Variable	OR^a^	95% CI	p-value	AOR^b^	95% CI	p-value
Sex						
Male	1.00			1.00		
Female	0.83	0.24–2.80	0.758	0.35	0.08–1.53	0.161
Educational level						
Primary or lower	1.00			1.00		
Higher than primary	2.45	1.41–4.26	0.001	1.57	0.85–2.93	0.151
Marital status						
Unmarried	1.00			1.00		
Married	1.66	0.85–3.30	0.140	1.26	0.60–2.67	0.541
Employment						
No	1.00			1.00		
Yes	1.08	0.62–1.90	0.777	0.61	0.31–1.20	0.151
Income per day	0.84	0.71–1.00	0.049	0.91	0.75–1.11	0.351
Shared needle at the last injection						
No	1.00			1.00		
Yes	1.87	1.00–3.51	0.051	1.45	0.73–2.91	0.290
Ever practiced flashblood						
No	1.00			1.00		
Yes	0.49	0.21–1.17	0.109	0.79	0.29–2.19	0.653
Ever had transactional sex						
No	1.00			1.00		
Yes	2.44	1.10–5.39	0.027	3.54	1.26–9.96	0.017
Anal sex in the past 6 months						
No	1.00			1.00		
Yes	0.83	0.34–2.02	0.689	1.06	0.37–3.03	0.918
Multiple partners						
No	1.00			1.00		
Yes	0.79	0.46–1.37	0.401	0.96	0.51–1.82	0.911
Condom use at last sexual encounter						
No	1.00			1.00		
Yes	3.28	1.90–5.65	<0.001	1.92	1.05–3.52	0.035
Polysubstance use in the past 30 days						
No	1.00			1.00		
Yes	0.76	0.46–1.27	0.299	0.84	0.47–1.50	0.561
History of imprisonment						
No	1.00			1.00		
Yes	1.10	0.62–1.93	0.743	1.53	0.80–2.94	0.202
Age*MAT^c^ program enrollment status						
Age	0.96	0.92–1.00	0.072	0.87	0.79–0.96	0.006
New enrollees	3.30	1.88–5.75	<0.001	0.03	0.001–1.23	0.064
Age*new enrollees	1.03	1.02–1.05	<0.001	1.14	1.02–1.27	0.020

^a^OR: odds ratio.

^b^AOR: adjusted odds ratio.

^c^MAT: integrated methadone-assisted treatment.

All variables were included in the final regression model.

### Factors associated with ever being screened for tuberculosis among participants

Table 6 shows the results of regression analyses. People who inject drugs were more likely to have ever been screened for tuberculosis when they had education levels higher than primary education (AOR: 1.77, 95% CI: 1.03–3.03), had a history of transaction sex (AOR: 4.35, 95% CI: 1.74–10.84), or had a history of imprisonment (AOR: 1.83, 95% CI: 1.09–3.08).

**Table 6:** Factors associated with ever being screened for tuberculosis among participants.

Variable	OR^a^	95% CI	p-value	AOR^b^	95% CI	p-value
Age	1.03	1.00–1.07	0.079	1.04	1.00–1.08	0.072
Sex						
Male	1.00			1.00		
Female	1.11	0.44–2.78	0.829	0.38	0.11–1.32	0.127
Educational level						
Primary or lower	1.00			1.00		
Higher than primary	2.11	1.31–3.42	0.002	1.77	1.03–3.03	0.039
Marital status						
Unmarried	1.00			1.00		
Married	1.49	0.82–2.69	0.187	1.32	0.69–2.54	0.399
Employment						
No	1.00			1.00		
Yes	1.09	0.68–1.73	0.730	0.83	0.46–1.50	0.545
Income per day	1.00	0.91–1.09	0.989	1.00	0.91–1.10	0.935
Shared needle at the last injection						
No	1.00			1.00		
Yes	1.77	1.03–3.04	0.038	1.25	0.69–2.30	0.461
Ever practiced flashblood						
No	1.00			1.00		
Yes	1.22	0.70–2.12	0.490	1.50	0.77–2.93	0.237
Ever had transactional sex						
No	1.00			1.00		
Yes	3.42	1.74–6.74	<0.001	4.35	1.74–10.84	0.002
Anal sex in the past 6 months						
No	1.00			1.00		
Yes	1.58	0.84–2.96	0.152	1.17	0.54–2.56	0.687
Multiple partners						
No	1.00			1.00		
Yes	0.73	0.47–1.16	0.184	0.63	0.36–1.11	0.113
Condom use at last sexual encounter						
No	1.00			1.00		
Yes	1.65	1.002–2.71	0.049	1.25	0.71–2.19	0.441
Polysubstance use in the past 30 days						
No	1.00			1.00		
Yes	0.88	0.58–1.34	0.547	0.84	0.51–1.37	0.492
History of imprisonment						
No	1.00			1.00		
Yes	1.64	1.04–2.57	0.032	1.83	1.09–3.08	0.022
MAT^c^ program enrollment status						
Community-recruited PWID^d^	1.00			1.00		
New enrollees	1.62	1.06–2.47	0.026	1.59	0.86–2.93	0.140

^a^OR: odds ratio.

^b^AOR: adjusted odds ratio.

^c^MAT: integrated methadone-assisted treatment.

^d^PWID: people who inject drugs.

All variables were included in the final regression model.

## Discussion

People who inject drugs engaged in high-risk behaviors but they poorly used screening services for common infectious diseases in Dar es Salaam, Tanzania. They engaged in multiple sexual partnerships, flashblood, sharing injecting needles, anal sex, and transactional sex. Despite these high-risk behaviors, only 36.0% of all participants had ever been screened for HIV infection, 18.5% for tuberculosis, 11.8% for any other STI, and 11.6% for hepatitis B/C.

The following factors were associated with ever being screened for infectious diseases. Those associated with increased rates of being screened for HIV infection included education levels above primary education, history of transactional sex, and being a new enrollee of the integrated methadone-assisted treatment program. The ones which were positively associated with ever being screened for hepatitis B/C screening were a history of transactional sex, a history of imprisonment, condom use at the last sexual encounter, being a new enrollee, and the interaction of being both a new enrollee and using condom at the last sexual encounter. Employment, however, was negatively associated with ever being screened for hepatitis B/C. A history of transactional sex and condom use at the last sexual encounter were positively associated with ever being screened for any other STI. Older age was negatively associated with ever being screened for any other STI. The interaction of being both older and a new enrollee, however, was associated with ever being screened for any other STI. Factors positively associated with ever being screened for tuberculosis were having education levels higher than primary education, a history of transactional sex, and a history of imprisonment.

In this study, participants practiced various high-risk behaviors. Various challenges promote these behaviors. The need for money forces some people who inject drugs to practice transactional sex as a source of income to obtain drugs [35]. Others share their blood (through flashblood) with colleagues who cannot buy drugs to reduce withdrawal symptoms [36]. Other challenges include intoxication with drugs and inability to negotiate condom use [37]. Some of them engage in high-risk behaviors because of drug dependence despite their knowledge of the associated risks [37].

People who inject drugs had poor access to screening services for infectious diseases in this study. Various barriers impair utilization of screening services among people who inject drugs. Low rates of HIV testing are a result of fear associated with knowing their HIV status, HIV and drug use related stigma, and a perception of low HIV infection risk [38–40]. In addition, low rates of STIs screening could be due to their asymptomatic nature during infection, making individuals not seek care [21]. Moreover, low hepatitis screening might be a result of their low awareness of the infection [25]. Efforts to overcome these barriers are necessary to improve utilization of screening services for infectious diseases among them.

In this study, people who inject drugs were more likely to have ever been screened for HIV infection or tuberculosis if they had education levels higher than primary education. Education is a fundamental social determinant of health [41]. Higher education has also been associated with better health outcomes [41]. Therefore, those who had that education level probably understood the importance of being screened for infectious diseases and sought for these services. For example, higher education attainment was associated with hepatitis C screening among people who inject drugs in the US [42].

A history of transactional sex was positively associated with ever being screened for all four infectious diseases. People who inject drugs engage in transactional sex to get money for drugs [37]. The Health Belief Model elucidates that individuals are more likely to seek for health care when they need it, if they have sufficient concern about their health [43]. Such individuals might have been concerned about their health due to their high-risk behaviors. Therefore they might have sought screening services to know their infection statuses.

A history of imprisonment was positively associated with ever being screened for hepatitis B/C and tuberculosis. Our data cannot verify that participants were screened for these infectious diseases in prisons. Our results, however, emphasize the importance of using this window of opportunity to provide screening services for infectious diseases to individuals in prisons to halt their transmission rates [44]. Similar to our study, HIV testing was positively associated with a history of incarceration among people who inject drugs in Russia [17].

Being a new enrollee of the integrated methadone-assisted treatment program was positively associated with ever being screened for HIV infection and hepatitis B/C. In a separate analysis, new enrollees were more likely to have higher education levels than primary education. Their higher likelihood of ever being screened for infectious diseases before they joined the program might be due to their higher education level, referring to the association of education with better health outcomes [40] as explained above.

Using condom at last sexual encounter was positively associated with ever being screened for hepatitis B/C or any other STI. Screening of infections diseases is provided with both pre- and post-test counseling of safer practices [45]. This counseling might have promoted the practice of safer sex. In the US, the use of condom increased among people who were using drugs after receiving voluntary counseling and testing of STIs [46]. Furthermore, the interaction of being both a new enrollee and using a condom at the last sexual encounter was positively associated with ever being screened for hepatitis B/C demonstrating the combined effect of the two factors on receipt of screening services.

Employment was, however, negatively associated with ever being screened for hepatitis B/C. People who inject drugs who had employment might have not been able to make time to seek for screening services. More research and exploratory qualitative research is needed to further understand this association. To this end, our result was different from that in the US where hepatitis C screening among people who inject drugs was not different between those employed and those who were not employed [42].

Older age was negatively associated with ever being screened for any other STI. The interaction of being both older and a new enrollee, however, was positively associated with ever being screened for any other STI. A separate analysis in this study showed fewer older community-recruited people who inject drugs had ever been screened for any other STI compared with new enrollees. The combined positive effect of the two factors on being screened probably was due to the higher education level [40] of new enrollees as explained above. However, more qualitative explanatory research is needed to understand these associations.

Two limitations should be noted while interpreting the results of this study. We recruited participants via convenience sampling which limits the generalizability of the study findings. While we recruited new enrollees from the hospital settings, we recruited people who inject drugs from the communities using snowball sampling owing to their hard-to-reach nature. Because these two sampling procedures were different, the participants in the two groups might have differences in the ability to access screening services for infectious diseases which we did not assess in this study. However, for the measures which we assessed, we adjusted for the participants’ differences in the regression analyses. Moreover, there is a risk of social desirability bias because the results are based on self-reported data. However, we tried to overcome this limitation by maintaining privacy during data collection.

In spite of the limitations, this study fills the gap of limited evidence about screening services for common infectious diseases among people who inject drugs in Tanzania. It examined their level of utilization and their associated factors in this key population. Results of this study are vital in the ongoing efforts to increase the coverage of interventions for people who inject drugs.

## Conclusions

People who inject drugs engaged in high-risk injecting and sexual behaviors, but they poorly used screening services for common infectious diseases in Dar es Salaam, Tanzania. They were more likely to have ever been screened for infectious diseases if they had higher education levels than primary education, were new enrollees of the integrated methadone-assisted treatment program, had a history of imprisonment, and the interaction of being both older and new enrollees. Our results suggest that, people who inject drugs are more likely to receive screening services for infectious diseases when they have higher levels of social integration. Outreach programs providing these screening services could improve rates of screening among people who inject drugs. In addition participants were more likely to have ever been screened for infectious diseases whether they practiced or did not practice high-risk behaviors. These findings highlight the need to increase the coverage of the screening services for infectious diseases for people who inject drugs, and strengthen the counseling of safer injecting and sexual practices to effect behavior change.

## Supporting Information

S1 Table Sociodemographic characteristics of participants stratified by integrated MAT program enrollment status (DOCX)

S2 Table Injecting and sexual behaviors of participants stratified by integrated MAT program enrollment status (DOCX)
