# Supplementary material for: A Mismatch between High-Risk Behaviors and Screening of Infectious Diseases among People Who Inject Drugs in Dar es Salaam, Tanzania
Source: PLoS One. 2016 Feb 5;11(2):e0148598. doi: 10.1371/journal.pone.0148598 (PMC4744010; doi:10.1371/journal.pone.0148598)
Supplement: S1 Table — (DOCX) [file pone.0148598.s001.docx]

**S1 Table: Sociodemographic characteristics of participants stratified by integrated MAT program enrollment status**

| **Variable** | **Total**  **(n = 578)** | **New MAT^a^ program enrollees (n = 273)** | **Community-recruited PWID^b^ (n = 305)** | **p value** |
| --- | --- | --- | --- | --- |
|  | **n (%)** | **n (%)** | **n (%)** |  |
| Age mean (SD^c^) | 34.6 (5.7) | 34.0 (6.7) | 35.1 (4.6) | 0.026 |
| Sex |  |  |  |  |
| Male | 548 (94.8) | 259 (94.9) | 289 (94.8) | 0.949 |
| Female | 30 (5.2) | 14 (5.1) | 16 (5.2) |  |
| Marital status |  |  |  |  |
| Married | 70 (12.1) | 62 (22.7) | 8 (2.6) | <0.001 |
| Unmarried | 508 (87.9) | 211 (77.3) | 297 (97.4) |  |
| Educational level |  |  |  |  |
| Primary or lower | 467 (80.8) | 184 (67.4) | 283 (92.8) | <0.001 |
| Higher than primary | 111 (19.2) | 89 (32.6) | 22 (7.2) |  |
| Employment |  |  |  |  |
| Yes | 408 (70.6) | 238 (87.2) | 170 (55.7) | <0.001 |
| No | 170 (29.4) | 35 (12.8) | 135 (44.3) |  |
| Income in US $ per day, mean (SD^c^) | 11.2 (14.9) | 7.9 (16.0) | 14.1 (13.1) | <0.001 |
| History of imprisonment |  |  |  |  |
| Yes | 152 (26.3) | 44 (16.1) | 108 (35.4) | <0.001 |
| No | 426 (73.7) | 229 (83.9) | 197 (64.6) |  |

^a^MAT: integrated methadone-assisted treatment.

^b^PWID: people who inject drugs.

^c^SD: standard deviation.
