# Supplementary material for: A Mismatch between High-Risk Behaviors and Screening of Infectious Diseases among People Who Inject Drugs in Dar es Salaam, Tanzania
Source: PLoS One. 2016 Feb 5;11(2):e0148598. doi: 10.1371/journal.pone.0148598 (PMC4744010; doi:10.1371/journal.pone.0148598)
Supplement: S2 Table — (DOCX) [file pone.0148598.s002.docx]

**S2 Table: Injecting and sexual behaviors of participants stratified by integrated MAT program enrollment status**

| **Variable** | **Total**  **(n = 578)** | **New MAT^a^ program enrollees (n = 273)** | **Community-recruited PWID^b^ (n = 305)** |  |
| --- | --- | --- | --- | --- |
|  | **n (%)** | **n (%)** | **n (%)** | **p-value** |
| Sharing needle at the last injection |  |  |  |  |
| Yes | 82 (14.2) | 48 (17.6) | 34 (11.2) | 0.027 |
| No | 496 (85.8) | 225 (82.4) | 271 (88.8) |  |
| Ever practiced flashblood |  |  |  |  |
| Yes | 90 (15.6) | 10 (3.7) | 80 (26.2) | <0.001 |
| No | 488 (84.4) | 263 (96.3) | 225 (73.8) |  |
| Ever had transactional sex |  |  |  |  |
| Yes | 39 (6.8) | 15 (5.5) | 24 (7.9) | 0.256 |
| No | 539 (93.2) | 258 (94.5) | 281 (92.1) |  |
| Anal sex in the past 6 months ^c^ |  |  |  |  |
| Yes | 58 (10.6) | 14 (5.6) | 44 (14.8) | 0.001 |
| No | 489 (89.4) | 235 (94.4) | 254 (85.2) |  |
| Multiple sexual partners ^c^ |  |  |  |  |
| Yes | 205 (37.5) | 62 (24.9) | 143 (48.0) | <0.001 |
| No | 342 (62.5) | 187 (75.1) | 155 (52.0) |  |
| Condom use at last sex ^c^ |  |  |  |  |
| Yes | 95 (17.4) | 79 (31.7) | 16 (5.4) | <0.001 |
| No | 452 (82.6) | 170 (68.3) | 282 (94.6) |  |
| Polysubstance use in the past 30 days |  |  |  |  |
| Yes | 323 (55.9) | 137 (50.2) | 186 (61.0) | 0.009 |
| No | 255 (44.1) | 136 (49.8) | 119 (39.0) |  |

^a^MAT: integrated methadone-assisted treatment.

^b^PWID: people who inject drugs.

^c^Among sexually active participants in the past six months, total n = 547.
